# The mitochondrial protease AtFTSH4 safeguards *Arabidopsis* shoot apical meristem function

**DOI:** 10.1038/srep28315

**Published:** 2016-06-20

**Authors:** Alicja Dolzblasz, Elwira Smakowska, Edyta M. Gola, Katarzyna Sokołowska, Marta Kicia, Hanna Janska

**Affiliations:** 1 Department of Plant Developmental Biology, Institute of Experimental Biology, Faculty of Biological Sciences, University of Wroclaw, Wroclaw, Poland; 2 Department of Cellular Molecular Biology, Faculty of Biotechnology, University of Wroclaw, Wroclaw, Poland

## Abstract

The shoot apical meristem (SAM) ensures continuous plant growth and organogenesis. In LD 30 °C, plants lacking AtFTSH4, an ATP-dependent mitochondrial protease that counteracts accumulation of internal oxidative stress, exhibit a puzzling phenotype of premature SAM termination. We aimed to elucidate the underlying cellular and molecular processes that link AtFTSH4 with SAM arrest. We studied AtFTSH4 expression, internal oxidative stress accumulation, and SAM morphology. Directly in the SAM we analysed H_2_O_2_ accumulation, mitochondria behaviour, and identity of stem cells using *WUS*/*CLV3* expression. AtFTSH4 was expressed in proliferating tissues, particularly during the reproductive phase. In the mutant, SAM, in which internal oxidative stress accumulates predominantly at 30 °C, lost its meristematic fate. This process was progressive and stage-specific. Premature meristem termination was associated with an expansion in SAM area, where mitochondria lost their functionality. All these effects destabilised the identity of the stem cells. SAM termination in *ftsh4* mutants is caused both by internal oxidative stress accumulation with time/age and by the tissue-specific role of AtFTSH4 around the flowering transition. Maintaining mitochondria functionality within the SAM, dependent on AtFTSH4, is vital to preserving stem cell activity throughout development.

Formation of all above-ground structures is possible throughout the life of a plant due to the indeterminate functioning of the shoot apical meristem (SAM). In *Arabidopsis thaliana*, the meristem has a typical tunica-corpus structure with two distinguishable outer layers (L1 and L2, tunica) and an internal layer (L3, corpus). The SAM is further organised into distinct zones that differ in their growth rate and gene expression patterns. The centre comprises a relatively constant pool of slowly dividing undifferentiated cells known as the stem cells, including the organising centre (OC) and initial cells^1^. In contrast, at the SAM periphery where organ primordia are specified, cells have a shortened cell cycle, higher divisional activity and a specific set of expressed genes [e.g. ref. 2]. Maintenance of the stem cells is necessary to provide sufficient new cells for tissue and organ formation, and is supported by the *WUS*/*CLV* feedback loop. *WUS*, expressed in the OC, keeps the distal stem cells undifferentiated while signalling from the stem cells via *CLV* genes negatively regulates *WUS* expression^1^,^3^,^4^,^5^.

The SAM initially produces rosette leaves, but during vegetative growth undergoes a transition from the juvenile to the adult stage, when plants become receptive to signals that trigger flowering. Concomitantly, during the transition to flowering, the adult vegetative SAM is transformed to the generative inflorescence SAM, which produces flowers and cauline leaves^6^. This switch is crucial to enable reproduction and is mediated by a genetic system modulated by both developmental and environmental cues [e.g. refs 7,8]. During these transitions, the SAM increases its proliferative activity, changes size, and for the flowering transition, it grows substantially upward^9^. Both transitions entail alterations in the pattern of gene expression and reorganisation of signalling pathways, but a general structural and functional continuity of the SAM is preserved. The generative inflorescence of *A. thaliana* formed through these processes is composed of multiple flowers arranged in a spiral, and is never topped by a terminal flower due to the presence of subtending lateral flowers^10^,^11^. The cessation of apical meristem growth (mitotic senescence) and the mechanisms of ongoing divisional arrest remain poorly understood^12^,^13^. Interestingly, one of the organelles involved in the senescence signalling are mitochondria.

Mitochondria structure and function must be coordinated and regulated [e.g. ref. 14]. It has been suggested that cell cycle arrest may be triggered by impaired mitochondrial function, which decreases their capacity to generate ATP^15^,^16^. While mitochondria of higher plants are commonly small and oval, the meristematic cells of the *Arabidopsis* SAM contain one large mitochondrion surrounding the nucleus alongside only a few small discrete mitochondria. These characteristics require dynamic architectural changes in mitochondrial shape, size, and distribution that likely relate to cell cycle-dependent functions such as mixing mtDNA and ensuring proper delivery of ATP during cell proliferation^17^.

Mitochondria are associated with reactive oxygen species (ROS), extremely reactive particles able to oxidise biological molecules [reviewed e.g. in^18^]. The amount of ROS is balanced through control of their formation and various antioxidant-scavenging systems, exemplified by the ascorbate peroxidases (APX) [e.g. ref. 19]. Redox regulation is similarly coordinated in the meristem. Jiang *et al*.^20^ have shown that the lengthened cell cycle of cells in the root quiescent centre (QC) results from their more oxidised state. It has also been shown that differences in superoxide and hydrogen peroxide levels can affect root growth and differentiation^21^ and that the regulation of several peroxidases impacts on root zonation pattern (meristematic vs. elongation zones), modulating the balance of ROS^22^.

Plant mitochondria play a central role in metabolism and various biosynthetic reactions to meet the structural demands of growth and development, in both sustaining and ending the life of a cell [reviewed e.g. in^23^]. As an important hub in cellular signalling and for ATP accessibility, they are important in determining whether resources are primarily used to maintain growth or are redirected for stress resistance, signalled by ROS [e.g. refs 24,25]. Mitochondria function is especially important during unfavourable conditions of growth: environmental stresses can cause oxidative damage by inducing increased ROS production as a consequence of perturbed metabolism, redox equilibrium and/or mitochondrial function [e.g. refs 26].

Mitochondria are one of the first targets of oxidative stress and involved in tolerance to it^31^, with morphology related to the cellular redox status. AtFTSH4 is an inner mitochondrial membrane-embedded metalloprotease that fulfils two contrasting functions, being both proteolytic and chaperone-like. It was shown that AtFTSH4 counteracts oxidative stress under short-day (SD), 22 °C conditions, probably by maintaining proper functionality of OXPHOS (oxidative phosphorylation) complexes and thus preventing ROS overproduction, and by removing oxidatively damaged proteins, which tend to be misfolded and form harmful insoluble aggregates. These conclusions were drawn from studies of *ftsh4* mutants, which exhibit a characteristic leaf phenotype under SD at 22 °C^32^,^33^,^34^. Zhang *et al*.^25^ have shown that the lack of AtFTSH4 causes the accumulation of hydrogen peroxide (H_2_O_2_), which deregulates auxin levels due to increased peroxidase activity affecting growth and development.

Direct data on redox regulation and mitochondria status in the SAM are surprisingly scarce, despite the significance of the SAM for continuous plant growth and development. In this study we analysed the phenotype of the premature arrested meristem in *ftsh4* mutants under the mild stress of elevated temperature (long day (LD), 30 °C). We investigate the impact of the internal oxidative stress on SAM maintenance by comparing wild-type (WT) and *ftsh4* mutant plants. We tested the following working hypotheses: (i) disturbed SAM activity in *ftsh4* is caused by internal oxidative stress; (ii) the stress accumulates progressively and in a stage-specific manner; (iii) H_2_O_2_ predominantly accumulates within the meristem of the mutants; and (iv) resultant mitochondria dysfunction occurs specifically in the SAM stem cells, ultimately causing premature meristem arrest.

## Results

### Effect of AtFTSH4 on plant development and reproduction

**Figure 1:**
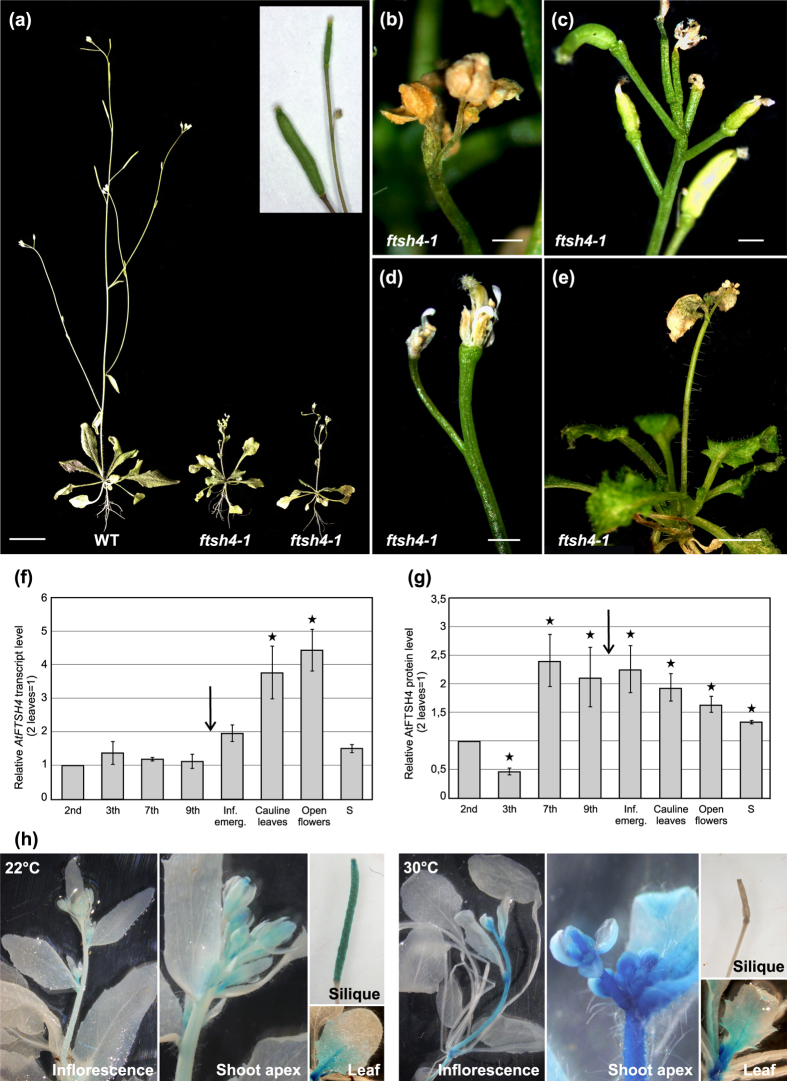
Expression analysis of *AtFTSH4* gene and *ftsh4* mutant phenotype. (**a**) The phenotypes of adult wild-type (WT) and *ftsh4* mutant plants grown under LD 30 °C. *ftsh4* mutants have a stunted main inflorescence stem, small organs, and no silique formation. Inset: inflorescence apex of adult WT plant just prior to growth cessation (rosette leaves are already dry) showing the last silique with a subtending flower bud. Scale bar: 15 mm. (**b**–**d**) Inflorescence apices of adult *ftsh4* mutants just prior to the growth cessation showing 3 features of axis termination: (**b**) a bunch of drying flower buds; (**c**) several flowers at the elongated pedicels with an aberrant arrangement; (**d**) a termination with two flowers. Siliques are not being formed in any case. Scale bars: 1 mm. (**e**) *ftsh4* mutant plant showing a characteristic growth cessation phenotype with drying apices of the main stem, when rosette leaves are still dark green. Scale bar: 5 mm. (**f**,**g**) The comparison of *AtFTSH4* transcript (**f**) and protein (**g**) level analysed during vegetative and generative development of WT plants grown under LD 30 °C. The collected tissues were: rosette leaves (2^nd^, 3^th^, 7^th^, 9^th^) sampled during the vegetative stage of development, 7^th^ leaf dissected after flowering (“infl. emergence”), cauline leaves, flowers and siliques. Abundance of *AtFTSH4* transcripts and protein is relative to the youngest tissue samples (2 leaves). Densitometric quantification is expressed as a ratio to the youngest tissue sample (2 leaves). Mean values ± SD from three experiments are shown. The arrow indicates the transition to flowering; the asterisks denote significant differences to the 2-leaf sample at p < 0.05. (**h**) Activity of the AtFTSH4 promoter visualised by the activity of the GUS reporter protein (blue). *pFTSH4:GUS* transgenic plants were grown under LD 22 °C and LD 30 °C and the expression level analysed during the generative stage of development. In the vegetative leaves, GUS activity accumulates similarly in both temperatures. After flowering, high GUS activity in plants grown at 30 °C is visible in actively growing tissues (shoot apices, stipules, flower buds, young internodes), but not in the siliques.

In both *ftsh4* mutant lines (*ftsh4-1* and *ftsh4-2*) under standard growth conditions of LD at 22 °C, no notable differences in morphology or development were observed, as previously reported^32^. However, under LD 30 °C, the mutants displayed striking phenotypic features related to generative development (Fig. 1a). The phenotypic abnormalities were very similar for both *ftsh4-1* and *ftsh4-2* mutant alleles (Supplementary Fig. 1a), and we focus only on the *ftsh4-1* line.

WT plants growing continuously in 30 °C formed inflorescences about 25 cm high, which terminated with a single silique with successive flower bud (Fig. 1a, and inset). In contrast, in plants lacking AtFTSH4 protease, inflorescence emergence was delayed and their stems were significantly shorter, reaching a maximum height of 6 cm (Fig. 1a). Inflorescence apices terminated either with several flower buds (max. 6), gathered at the tip of the axis (23.8% of flowering plants, Fig. 1b), or with a few separate, fully developed flowers on elongated pedicels (76.2% of flowering plants), lacking successive flower buds or a subtended leaf (Fig. 1c,d). The first symptom of growth termination was abrupt drying of the flowers and the tips of cauline leaves (Fig. 1e). In WT plants, on the other hand, it is the vegetative rosette that tends to senesce and dry first, while the inflorescence tip has still visible flower buds (Fig. 1a, inset). Importantly, *ftsh4* mutants at 30 °C were not able to produce siliques and therefore no seeds were formed, impairing reproduction of mutant plants. Notably, WT plants grown at 30 °C had shortened siliques and thus a lower total seed number compared to WT plants grown at 22 °C. WT plants cross-pollinated with 22 °C WT pollen developed normal siliques (Supplementary Fig. 2), whereas silique development in cross-pollinated *ftsh4* mutants was not improved, resulting in drying carpels (Supplementary Fig. 2b). These results show that mutant plants grown in 30 °C conditions are not only defective in pollen production but also are female sterile.

### Temperature effects on AtFTSH4 expression

*AtFTSH4* expression at 30 °C remained mostly constant with a pronounced increase after floral transition, especially in generative organs like cauline leaves and flowers (Fig. 1f). To visualise the spatial responsiveness of the AtFTSH4 promoter to stress at 30 °C in the generative phase, we quantified the activity of the AtFTSH4-promoter-driven *GUS* (*β*-glucuronidase). Under LD 22 °C, the *GUS* reporter was expressed predominantly in the meristematic cells of the shoot apex, the neighbouring youngest tissues and in siliques (Fig. 1h). At 30 °C, the reporter gene was expressed in the same tissues but its level increased markedly (Fig. 1h), what was confirmed also at the *AtFTSH4* transcript level (Supplementary Fig. 1b). Interestingly, at the elevated temperature, mature siliques displayed relatively weak activity of the *pAtFTSH4*:*GUS* transgene compared to at 22 °C. Importantly, in both growing conditions, expression decreased in association with organ maturation, acropetally in internodes and basipetally in leaves (Fig. 1h). In parallel with the increased level of transcript, Western blotting showed significantly elevated amounts of AtFTSH4 protein in cauline leaves and flowers of WT plants grown at 30 °C (Fig. 1g) comparing to the youngest developmental stage (2 leaves). Interestingly, this protein abundance was already evident in the last leaves of the adult phase, prior to inflorescence emergence (Fig. 1g). As we observe the shift in the maximum between upregulation at the transcriptional and protein level it is highly probable that AtFTSH4 undergoes stage specific stabilization in 30 °C at the protein level.

### Variability in the phenotypic response of AtFTSH4 related to temperature regime

WT and mutant plants were transferred from standard 22 °C to complete their development at an elevated temperature of 30 °C at one of the four developmental stages: two leaves, juvenile (4^th^ leaf), adult (6/7^th^ leaf), or after transition to flowering. A parallel experiment was performed to examine the ability of SAM to recover after stress, in which plants were transferred from the elevated temperature of 30 °C to standard 22 °C at the same developmental stages. Control plants were continuously grown either at 22 °C or 30 °C. In both experiments, SAM activity was measured as the main inflorescence height and the amount of flowers formed, and plant reproductive ability was measured in terms of silique formation.

**Figure 2:**
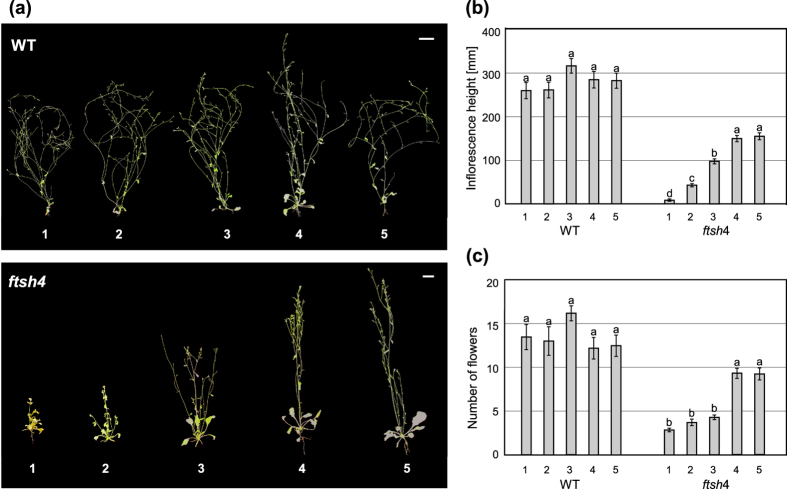
The cumulative effect of continuous mild stress of 30 °C on the phenotype of *ftsh4* mutants. (**a**) WT and *ftsh4* plants showing different phenotypes depending on the timing of transfer from LD 22 °C to 30 °C: (1) grown continuously at 30 °C, (2) two leaves, (3) juvenile, (4) adult, (5) after bolting. *ftsh4* mutants’ ability to maintain the inflorescence meristem gradually decreases with time spent at 30 °C, indicated by the main stem height. Wild-type plants always have the same phenotype. Scale bars: 1 cm (**b**,**c**) The comparison of the inflorescence shoot activity assessed by (**b**) the height of the main stem and (**c**) the number of flowers of WT and *ftsh4* mutant plants, depending on the timing of transfer from LD 22 °C to LD 30 °C. Main inflorescence height decreases, as does the number of flowers in the *ftsh4* mutants. Mean values ± SE are shown. Significant differences between bars at p < 0.05 are denoted by different lower case letters.

In the first experiment, WT plants transferred to 30 °C had a similar height and number of flowers to controls, and in all experimental conditions, they were able to form siliques (Fig. 2). In contrast, *ftsh4* mutants had shorter inflorescences the earlier they were transferred to the elevated temperature (Fig. 2a,b). The number of flowers was similar in plants transferred at the 2-leaf and juvenile stages, but greatly reduced in comparison to WT plants. Plants transferred at the adult stage or later were much less affected (Fig. 2a,c). Importantly, all *ftsh4* plants grown at 30 °C (n = 25) as well as those transferred to 30 °C at the 2-leaf (n = 15) and juvenile (n = 67) stages did not form any siliques. *ftsh4* plants transferred at the adult stage or after bolting were able to form siliques only in 38% (n = 63) and 64% (n = 63) of cases, respectively.

**Figure 3:**
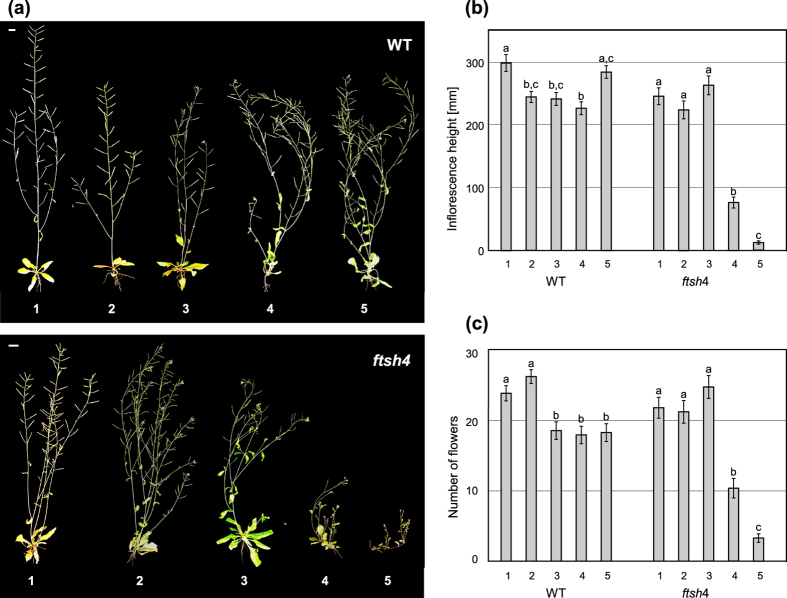
Meristem recovery after mild temperature stress. (**a**) Wild-type and *ftsh4* plants showing a different phenotype depending on the timing of transfer from LD 30 °C to LD 22 °C: (1) grown continuously at 22 °C, (2) two leaves, (3) juvenile, (4) adult, (5) after bolting. *ftsh4* mutants show a decreased ability to maintain the inflorescence meristem, as judged by the main stem height, the longer they grew at 30 °C. Scale bars: 1 cm. (**b**,**c**) The comparison of the main inflorescence activity assessed by (**b**) the height of the main stem and (**c**) the number of flowers of wild-type and *ftsh4* mutant plants depending on the timing of transfer from LD 30 °C to LD 22 °C. Meristem activity is maintained until plants are subjected to the stress during the adult phase (committed to flowering). Mean values ± SE are shown. Significant differences between bars at p < 0.05 are denoted by different lower case letters.

In the second experiment, WT plants transferred from 30 °C to 22 °C showed only small developmental differences (Fig. 3). *ftsh4* mutants transferred before the adult stage also showed no reduction in the number of flowers or inflorescence height compared to WT (Fig. 3). In contrast, in mutants transferred at the adult stage (just prior to bolting) or after bolting, main stem development was terminated almost immediately (Fig. 3b,c). Interestingly, after several weeks, only plants transferred at the adult stage were able to recover, producing up to 8 axillary stems with flowers and siliques (Supplementary Fig. 3).

These results show that SAM indeterminacy is lost progressively, and in addition the defect becomes further enhanced around the flowering transition at 30 °C.

### Internal oxidative stress accumulation in the SAM

**Figure 4:**
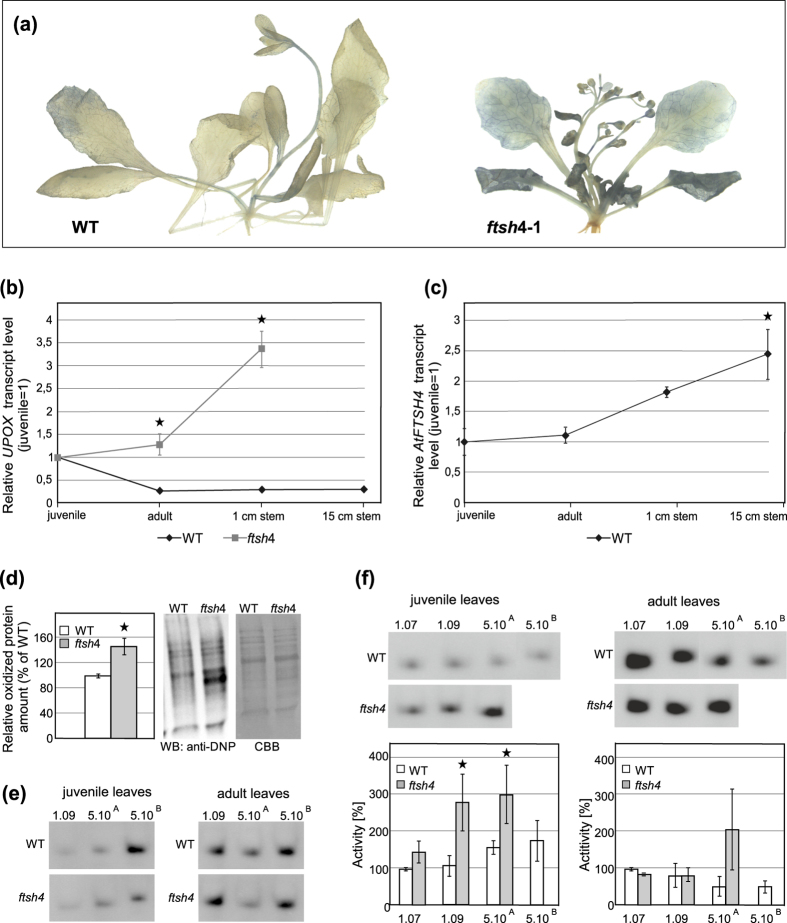
Internal oxidative stress accumulation in *ftsh4* mutant plants grown at 30 °C. (**a**) NBT assay showing the accumulation of superoxide radicals O˙^**−**^ (dark blue colour) in adult and flowering wild-type and *ftsh4* mutant plants. (**b**) Expression level of the oxidative stress marker, *UPOX*, in the shoot apices (SAM and the youngest primordia), measured in different developmental stages: juvenile, adult, 1 cm high young flowering plant, and additionally for the WT also mature (c. 15 cm high) plants grown at 30 °C. Transcript levels for gene encoding UPOX in *ftsh4* mutants are shown relative to WT plants. Mean values ± SD from three experiments are shown. Asterisks denote significant differences from the juvenile sample at p < 0.05. (**c**) The expression level of the *AtFTSH4* gene in the shoot apices (SAM and youngest primordia) of WT plants, measured in different developmental stages: juvenile, adult, 1 cm high young flowering plant, and mature (c. 15 cm high) plants grown at 30 °C. Mean values ± SD from three experiments are shown. Asterisks denote significant differences from the juvenile sample at p < 0.05. (**d**) Immunodetection with anti-DNP antibodies and quantification of carbonylated proteins in total protein extracts from shoot apices (SAM with the youngest primordia), separated by one-dimensional gel electrophoresis. The amount of oxidised protein was calculated as a percentage of the amount in WT plants. Mean values ± SD from three experiments are shown (left). Asterisks denote significant differences at p < 0.05. On the right, a representative picture after gel electrophoresis is shown. (**e**,**f**) The activity of ascorbate peroxidases (APX) measured in juvenile and adult leaves at different age of *ftsh4* and WT plants grown at (**e**) 22 °C and (**f**) 30 °C. Developmental stages are as follow: 1.07 – when the 7^th^ leaf is present, 1.09 – when the 9^th^ leaf is present, 5.10^a^ – after bolting (inflorescence up to 1 cm), and 5.10^b^ – mature inflorescence. APX activity at 30 °C was quantified as a percentage of the WT activity level. Mean values ± SD from three experiments are shown (**f**, below). Asterisks denote statistical significance.

In *ftsh4* mutants, superoxide (O˙^−^) radicals accumulated especially in the last-formed adult vegetative leaves and in generative structures (Fig. 4a). In comparison to WT plants, *UPOX* gene (gene up-regulated by oxidative stress) expression significantly increased in all organs of the *ftsh4* mutant, particularly in the generative structures (Supplementary Fig. 4). In addition, the *UPOX* transcript level in the SAM itself (with the youngest primordia) was analysed in plants at different developmental stages: juvenile, adult and after bolting (1 cm stem), and, for WT, in older inflorescences (around 15 cm stems). It did not change in developing WT plants, whereas in *ftsh4* mutants it accumulated with age. The increase was even more prominent after bolting (Fig. 4b). The accumulation of *UPOX* with time correlates well with the increased mRNA abundance of *AtFTSH4* in aging shoots of WT plants (Fig. 4c). The generative SAMs of the *ftsh4* mutant accumulated 40% more carbonylated proteins, one of the most striking symptoms of severe oxidative stress, in comparison to WT plants (Fig. 4d).

The activity of APX enzymes under standard 22 °C conditions increased with age in the juvenile leaves of both genotypes. In the adult leaves, however, it decreased after the floral transition, and then increased again in the older plants (Fig. 4e). Thus, organs formed at different developmental stages differ in their antioxidant response, in similar ways in both WT and *ftsh4*. In the juvenile leaves of plants growing at 30 °C, APX activity was consistently up-regulated. The same pattern was evident at 22 °C, though more pronounced in the *ftsh4* mutants, at all developmental time-points (Fig. 4f). Interestingly, in the adult leaves, APX activity did not differ significantly between WT and *ftsh4* mutants. This shows that at 30 °C, the response to accumulating stress differs depending on the developmental phase.

**Figure 5:**
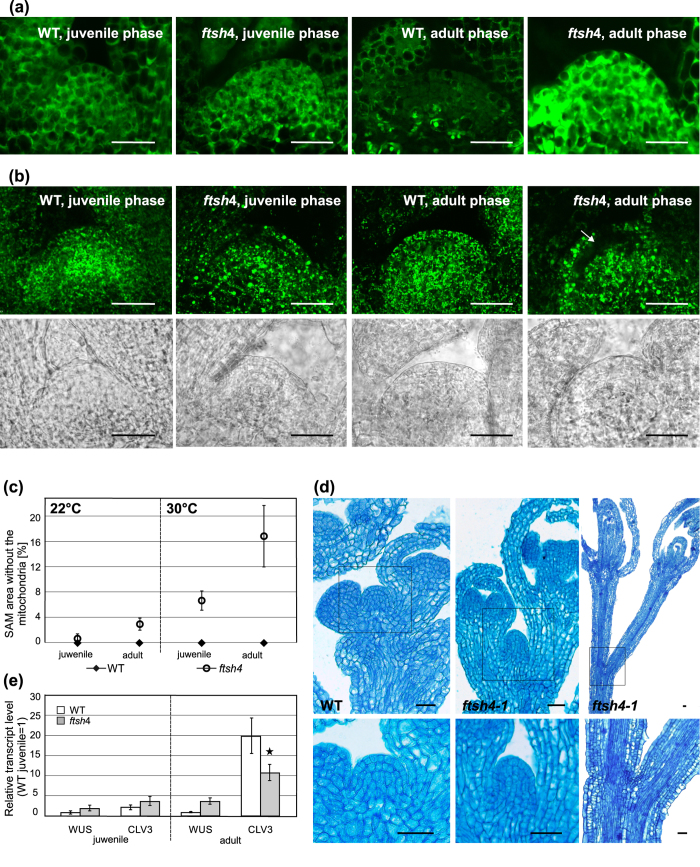
Meristem structure and stress response of *ftsh4* mutants grown at 30 °C. (**a**) The comparison of *in vivo* H_2_O_2_ accumulation in the meristems of juvenile and adult wild-type and *ftsh4* mutants grown at 30 °C. Increased signal from the probe was detected only in adult *ftsh4* mutant plants. Scale bars: 25 μm. (**b**) Distribution of mitochondria and their morphology *in vivo* in the meristems of juvenile and adult wild-type and *ftsh4* transgenic lines grown at 30 °C. WT plants (WT;35S:Mt-GFP) are characterised by small mitochondria evenly distributed throughout the meristem, whereas in *ftsh4* mutant plants (*ftsh4*;35S:Mt-GFP) mitochondria aggregate and some areas of the meristem completely lack GFP signal (denoted with arrow). The bright-light images of the corresponding meristems are presented below. Scale bars: 25 μm. (**c**) The average area (%) of the SAM with no GFP signal in the mitochondria in juvenile and adult wild-type and *ftsh4* mutant plants grown at 22 °C and 30 °C. Only *ftsh4* mutants have areas without GFP signal. Mean values ± SD from three experiments are shown. (**d**) The comparison of WT and *ftsh4* mutant meristem structures at the generative stage prior to growth cessation. WT always had the same meristem structure prior to growth termination and the *ftsh4* mutant plants showed 2 different types of growth cessation: normal dome-shape meristem, and no meristem. The upper panel presents the general overview of the meristems, and the lower panel represents their magnifications. Scale bars: 25 μm. (**e**) Expression levels of *WUS* an*d CLV3* in the shoot apices (SAM and youngest primordia) of the WT and *ftsh4* plants, measured in juvenile and adult stages grown at 30 °C. Mean values ± SD from three experiments are shown. At both developmental stages the reference level was from the WT juvenile samples. Asterisks denote significant differences between WT and *ftsh4* mutants in a given developmental stage.

Next, to investigate differences in the H_2_O_2_ level in *ftsh4* mutants vs. WT plants, the CH-H_2_DCFDA probe was applied to the dissected vegetative meristems of juvenile and adult plants of both genotypes grown at 30 °C. In the SAM of juveniles, the signal of the CH-H_2_DCFDA probe for H_2_O_2_ was relatively weak in both genotypes (Fig. 5a) and did not differ substantially. By comparison, the level of the H_2_O_2_ marker was higher in the SAM of adult *ftsh4* mutant plants (Fig. 5a), clearly indicating that internal oxidative stress accumulates directly in the adult meristem.

### Mitochondria distribution in the SAM

**Figure 6:**
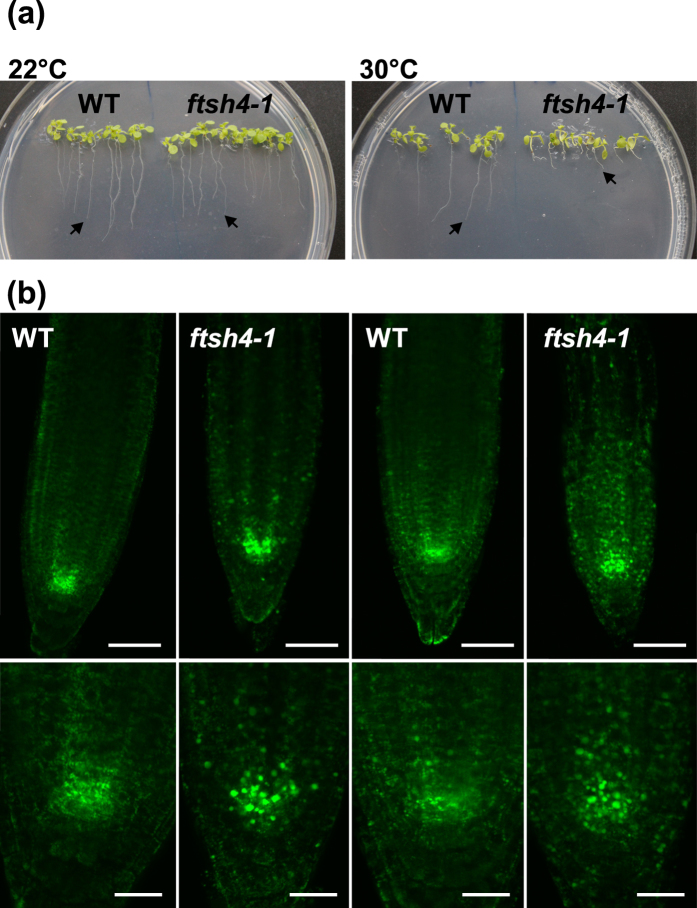
Root meristem arrest of *ftsh4* mutants grown at 30 °C. (**a**) Root phenotype of wild-type (WT) and *ftsh4* mutant plants grown at 22 °C (left picture) and at 30 °C (right picture). (**b**) Distribution of mitochondria and their morphology *in vivo* in the meristems of WT and *ftsh4* transgenic lines grown at 22 °C (at left) and 30 °C (at right). WT plants (WT;35S:Mt-GFP) are characterised by small mitochondria evenly distributed throughout the meristem, whereas in *ftsh4* mutant plants (*ftsh4*;35S:Mt-GFP) giant mitochondria can be found. The upper panel presents the general overview, and the lower panel represents their magnifications. Scale bars: 20 μm (upper pictures), 50 μm (lower pictures).

In order to investigate mitochondria, vegetative SAM was dissected by hand from at least 10 transgenic WT and 10 *ftsh4-1* mutant plants (WT or *ftsh4-1*;35S:Mt-GFP) and directly analysed in confocal laser microscopy. In juvenile and adult WT transgenic plants (WT;35S:Mt-GFP) from both growth conditions, evenly distributed, small, discrete mitochondria were visible (Fig. 5b, Supplementary Fig. 4). In contrast, some mitochondria in transgenic *ftsh4* plants (*ftsh4*;35S:Mt-GFP) had an aberrant morphology, in accordance with our recent finding (personal communication, R. Skibor). Interestingly, we found that mitochondria within the mutant SAM were surprisingly unevenly distributed at both temperatures compared to WT plants (Fig. 5b, Supplementary Fig. 4). Mutant plants grown in 30 °C showed a strikingly large region of meristem devoid of the fluorescent GFP signal from mitochondria. The first signs of mitochondria impairment were visible in the SAM in juveniles (around 6.5% of the SAM area; Fig. 5b,c), which increased significantly with the age to reach around 17% of the total area of adult SAM (Fig. 5b,c). This SAM area consisted mostly of L2 and occasionally L1 and L3 layers. The centre of the meristem, where initial cells are located, was most strongly affected. At 22 °C, the regions without mitochondrial signal occupied only a comparatively small area of the juvenile (<1%) and adult (approximately 3%) vegetative SAM, also showing no significant increase with age in the *ftsh4* mutant (Fig. 5c, Supplementary Fig. 4). Importantly, in the *ftsh4* mutant, cells from the SAM region with impaired mitochondria still have normal morphology, nuclei, and proplastids (Fig. 5b, Supplementary Fig. 4). In WT plants, no SAM regions were observed that lacked the GFP signal (Fig. 5b,c, Supplementary Fig. 4). The presence of giant mitochondria among healthy ones was also observed in the RAM (Root Apical Meristem) of 8 day old *ftsh4* plants, when grown in both LD, 22 °C and LD, 30 °C. In contrary to the SAM, no area without a visible signal from the GFP was ever found in the examined roots (Fig. 6).

Together, these findings strongly suggest that the loss of AtFTSH4 affects the morphology of mitochondria, but their distribution is altered only in the SAM.

### Meristem maintenance in the *ftsh4* mutant

The observed impairments within the SAM prompted us to examine its structure during growth at 30 °C. The meristem of the *ftsh4* mutants in both the juvenile and adult vegetative stages was comparable to that in the WT plants and presented a typical layered organisation (Supplementary Fig. 4). In the generative stage of *ftsh4* mutants, two different organisations of the inflorescence apices occurred: (a) a normal dome-shaped meristem, as in WT plants, and (b) completely lacking a meristem and terminating with 2 flowers on elongated pedicels (Fig. 5d). Dome-like meristems of *ftsh4* mutant plants had a typical organisation with proper L1 and L2 layers (Fig. 5d).

In the juvenile meristems, *WUS* and *CLV3* genes were expressed comparably in both WT and *ftsh4* mutant plants (Fig. 5e). Adult vegetative meristems of the WT plants showed a strong increase in the expression of the *CLV3* gene, in comparison to the juvenile meristems. Interestingly, *ftsh4* plants were characterised by much less pronounced increase of *CLV3* gene, when compared to WT (Fig. 5e). In contrast, *WUS* levels did not change significantly between the developmental stages, nor between the two genotypes (Fig. 5e). These results suggest that the ability to maintain the identity of the stem cells in the mutant plants, in 30 °C, decreases with age in parallel with the progressive mitochondria impairment.

## Discussion

In this study, we have analysed the impact of mitochondria impairment, caused by a loss of the mitochondrial protease AtFTSH4, on the maintenance of the structural and functional integrity of the SAM. We found that *ftsh4* mutants abruptly terminate shoot axis growth after the transition to flowering in suboptimal growth conditions (continuous mildly elevated temperature of 30 °C). Axis growth ceases either without significantly altered meristem organisation, as shown by its normal shape and layering, or by complete meristem disappearance due to meristematic cells being diverted to form flower primordia. Our results indicate that the SAM initial cells, which must be maintained throughout the plant’s life, lose the functionality of their mitochondria in *ftsh4* mutants, which causes a drop in the cells’ meristematic activity around the time of flowering. We postulate that the mitochondrial protease AtFTSH4 safeguards the SAM function in at least three interrelated ways: progressively, stage- and SAM-dependent.

Transcript and protein levels of the AtFTSH4 protease demonstrated that its importance increases progressively through development in *Arabidopsis*, being most prevalent just prior to and during the generative stages of growth. *AtFTSH4* gene promoter activity, visualised by the *GUS* reporter, further supported its requirement for the generative growth as well as showing its prominence in the meristematic tissues. The progressive and stage-specific role of AtFTSH4 was further confirmed by experimental results showing that the earlier the mutants were transferred from 22 °C to 30 °C, the quicker the SAM was terminated. However, the number of flowers did not reduce in the same gradual way, suggesting the involvement of not only for the time duration but also the stage at which plants are exposed to temperature stress. In the complementary experiment (transfer from 30 °C to 22 °C), the SAM of the mutants fully recovered from the stress without affecting the final growth rate if the elevated temperature did not persist until the adult phase. These results support our conclusion that AtFTSH4 plays a progressive and stage-specific role, being particularly important for SAM function around flowering time.

Quantification of oxidative stress showed that the vegetative meristems of *ftsh4* plants gradually accumulate oxidative stress only in the adult, and not in the preceding juvenile stage. Internal oxidative stress is further accelerated after the subsequent transition to generative development. Even though the dissected shoot apices in which *UPOX* expression and protein carbonylation were quantified may have contained other tissues than meristem, they were certainly strongly enriched with SAM tissues. Notably, the fluorescence-based *in vivo* H_2_O_2_ detection within the SAM clearly proved that ROS accumulated directly in the adult vegetative meristem.

At 30 °C, APX increased markedly with time only during the juvenile phase, not in the adult phase. Thus, the stage-dependent susceptibility of *ftsh4* plants to elevated temperature during vegetative growth may be due to differential activity of the antioxidant system. Furthermore, Johansson *et al*.^35^ have shown that protein carbonylation, a consequence of oxidative stress, increases with age in WT *Arabidopsis* plants and drops abruptly just prior to flowering. Eliminating oxidatively damaged proteins in this way seems to be crucial to ensure fitness of offspring, and this process is mediated by AtFTSH4 at that particular time.

We have shown that abrupt meristem growth cessation in *ftsh4* in 30 °C is not only due to the absence of progressive and stage-specific roles of the AtFTSH4, but is additionally triggered by a lack of its specific function within the SAM. The adult vegetative meristems of transgenic *ftsh4* plants with labelled mitochondria had distinct regions within their SAM that lacked the strong GFP fluorescent signal. These regions were present regardless of growth conditions, but significantly expanded with age only at 30 °C. Our results indicate that the driving force for the expansion of the region of reduced mitochondrial activity is the accumulation of internal oxidative stress in mutants growing at 30 °C, which is specific to the adult vegetative stage. Thus, all roles of AtFTSH4 described here – SAM-specific, progressive, and stage-dependent – interconnect and enhance each other at 30 °C. It should be emphasised here that the cells in the region without visible mitochondria possess seemingly normal cell morphology in relation to other organelles, such as nuclei and proplastids. The lack of a strong GFP signal localised in mitochondria therefore rather does not reflect the absence of mitochondria, but in our opinion is a consequence of decreased membrane potential and hence impaired import of GFP fusion protein in these organelles. Unfortunately, as stem cells of the SAM are difficult to handle *in vivo*, we were not able to determine the sequence of events in more detail. It is important to stress out that, around the time of flowering, the affected region encompassed the whole or part of the stem cell zone within the meristem, where the unaffected initial cells have to be preserved throughout the plant’s life. Interestingly, such an aberration is not a feature characteristic of all meristematic tissues, but is highly specific for the SAM.

We hypothesise that the underlying mechanism by which AtFTSH4 affects membrane potential exclusively in the stem cell zone is associated with the unique redox status of these cells. Data on the other plant apical meristem, the root tip, support this notion, as the central cells from the quiescent centre (QC) were shown to be of more oxidised redox status than the neighbouring cells^20^. More oxidised QC status correlated with decreased membrane potential in the mitochondria, and was responsible for reduced divisional activity of the cells and reduced capacity of the mitochondria to generate ATP. Interestingly, the redox status of the QC was influenced by the hormone auxin^20^. The interplay between auxin and cytokinin determines the stem cell proliferation/differentiation balance, regulating SAM size and activity and modulating meristematic gene expression^36^,^37^,^38^,^39^,^40^,^41^,^42^,^43^. A link between AtFTSH4 activity, auxin homeostasis and redox status has previously been suggested^25^. AtFTSH4 down-regulates peroxidases, preventing indole-3-acetic acid oxidative decarboxylation^25^. It is therefore plausible that disturbed homeostasis and signalling of auxin and/or other hormones is at least partially responsible for the *ftsh4* SAM termination at 30 °C. The redox status of the SAM stem cells, which must be very carefully balanced, would then be affected first.

In line with the decreased mitochondria functionality in the mutant, we found that the expression level of the stem cell marker *CLV3* decreased in the adult vegetative stage compared to the WT plants. Thus, the identity of the initial cells is already compromised prior to flowering. At the same time, *WUS* expression tended to increase in the mutants, indicating a lack of negative regulation from the stem cells. This accords with the observation that mitochondria in the OC cells in the L3 layer mostly remain unaffected. As a consequence, at 30 °C, SAM cell homeostasis and function in *ftsh4* mutants is disturbed, which leads to precocious axis termination. Interestingly, in 30 °C the ratio between *CLV3* and *WUS* genes expression, and subsequently the SAM size, increases in WT plants. It is plausible that to support that bigger requirements for the regulation of the stem cells WUS protein is more effective. In contrast, the reduced *CLV3* and maintained similar *WUS* expressions in *ftsh4* mutant background, comparing to WT, suggest inadequate WUS activity. As a consequence, at 30 °C, SAM cell homeostasis and function in *ftsh4* mutants is disturbed, which leads to precocious axis termination. It is also conceivable that in the mutant, the excessive number of SAM cells, and perhaps even organ primordia cells, become quiescent and so the axis growth and development terminates.

To summarise, the precocious shoot axis arrest that is the most prominent phenotype of the *ftsh4* mutant arises from the SAM-specific role of AtFTSH4. That SAM-specific function is enhanced by progressive- and stage-dependent accumulation of internal oxidative stress, resulting in the expansion of the SAM area in which mitochondria lose their membrane potential, specifically in the stem cells. In conclusion, the AtFTSH4 protease specifically safeguards SAM function by preventing mitochondrial oxidative damage within the initial cells. AtFTSH4 has a specific role in the meristem, mainly at the flowering transition stage, which can be related to: (1) eliminating oxidised proteins, particularly in the SAM, to improve fitness of the next generation; (2) balancing hormonal homeostasis of the SAM; (3) regulating ROS levels and mitochondria membrane potentials in different cell types of the SAM; (4) sustaining resources, e.g. the ATP supply, for growth demands and divisions, which increase at the flowering transition. It would be profitable to explore cell divisional activity and hormonal interplays to fully understand the processes that lead to SAM arrest.

## Methods

### Plant material and growth conditions

*Arabidopsis thaliana* (L.) Heynh. Columbia-0 (Col-0) was used as the WT reference. The previously characterised transgenic lines *ftsh4-1* (SALK_035107/TAIR) and *ftsh4-2* (GABI_103H09/TAIR)^32^ were obtained from the Salk Institute and the Max Planck Institute for Breeding Research, respectively, and the DIPS*-GFP* reporter line (*GFP* gene fused to the mitochondria targeting the δ-ATPase sequence under promoter CaMV35S) from W. Sakamoto^44^. Plants were grown in a 16 h light/8 h dark (long day, LD) photoperiod at 22 °C and 30 °C.

### Vector construction, transformation, and GUS staining

To construct the *pAtFTSH4:GUS* reporter line, the 739-nucleotide regulatory sequence upstream of the *AtFTSH4* gene, which contains its signal peptide, was selected based on *in silico* predictions using PPDB (Plan Promoter Database; http://ppdb.agr.gifu-u.ac.jp/ppdb/cgi-bin/index.cgi) and AGRIS (*Arabidopsis* Gene Regulatory Information Server; http://arabidopsis.med.ohio-state.edu). This promoter region was cloned into the pCAMBIA1304 binary vector and introduced into *Agrobacterium tumefaciens* (strain LBA4404) by electroporation, which was used to transform the *A. thaliana* (Col-0) plants by floral dip vacuum infiltration^45^. The stable integration of T-DNA into the genome of the transgenic plants (*pAtFTSH4:GUS*) was verified via genomic PCR analysis using *GUS* gene-specific primers.

*GUS* gene activity, under the control of the AtFTSH4 promoter, was analysed in *Arabidopsis* plants grown in LD/22 °C and LD/30 °C at the generative stage of their development. Tissues were prefixed in ice-cold 90% (v/v) acetone for 30 min at room temperature and then rinsed with 50 mM sodium phosphate buffer (pH 7.2). Staining was performed using 2 mM X-gluc (5-bromo-4-chloro-3-indolyl ß-d-glucuronide cyclohexamine) for 16 h in the dark at 37 °C^46^,^47^. Tissues were then treated with increasing ethanol concentrations, fixed with FAA solution (50% ethanol, 3.7% formaldehyde, 5% acetic acid) and stored in ethanol. Images were obtained using a stereomicroscope (see *Microscopy* section).

### Histological analyses

To visualise SAM structures, shoot apices from the *ftsh4*-1 mutant and WT were dissected from juvenile vegetative, adult vegetative, and flowering plants (more than 20 plants for each genotype and for each developmental phase). Plant material was fixed in FAA solution, dehydrated in a tertiary butyl alcohol series and embedded in Paraplast X-tra. Series of longitudinal sections 6–10 μm thick were cut on a rotary microtome and doubled-stained with Alcian Blue-Safranin O solution (1:2 v/v)^48^. To visualise nuclei within the SAM, sections were stained with 5 mg/mL Hoechst 33342 solution. Histochemical detection of superoxide radicals (O˙_2_^−^) was performed by infiltrating living, excised plants with 1 mg/mL nitroblue tetrazolinum (NBT) solution^49^. The SAM structure and different staining reactions were documented using a stereomicroscope and an epi-fluorescence microscope (see *Microscopy* section).

### Real-time PCR

Real-time PCR analyses were performed using a LightCycler 2.0 and Real-Time 2× PCR Master Mix SYBR version B. The total reactions volume was 15 μL with a final primer concentration of 0.5 M. Material from a pool of WT plants served as the calibrator, using the *PP2AA3* gene (At1g13320) as a reference. Amplification efficiency for each analysed amplicon was calculated based on standard curves from serial 2-fold dilutions of the cDNA samples. The amplification protocol comprised: denaturation, 95 °C for 1 min; amplification, 45 cycles at 95 °C for 10 s, 55–65 °C (primer-specific annealing temperature) for 10 s, 72 °C for 20 s with single data acquisition; cooling, 40 °C for 30 s. The specificity of the amplification products was verified by melting curve analysis. Real-time PCR analyses were performed on at least three independent biological repetitions. The primers used are listed in Supplementary Table S1. Differences in the level of transcript between developmental stages were tested using t-tests.

### Total protein extraction

Total protein extract was obtained simultaneously from WT and *ftsh4* plants as described by Martinez-Garcia *et al*.^50^. Protein concentration was determined using the DC Protein Assay.

### Immunoblot Analysis

Equal amounts of proteins from wild-type (WT), at annotated in Fig.1g developmental stages, (25 μg per line) were separated by SDS-PAGE according to Laemmli^51^. After electrophoresis, proteins were transferred to PVDF membrane and immunostained with ant-AtFTSH4 (AS07 205) antibody purchased from Agrisera, Umea, Sweden. Immunodetection was performed using the WesternBrightTM Quantum Western Blotting Detection Kit (Advansta, Menlo Park, CA, USA) and the optical density of the bands was quantified using the Image Quant software (Molecular Dynamics, Sunnyvale, CA, USA).

### Detection of Carbonylated Proteins on One-Dimensional gels

Carbonylated proteins were isolated using derivatization of protein carbonyl groups with 2,4-dinitrophenylhydrazine (DNPH) in the OxyBlot kit (Millipore) and detected using immunodetection. This was performed with a primary antibody directed against dinitrophenylhydrazone, using 25 μg per lane of total protein extract from *ftsh4-1* and WT plants. The carbonylated proteins were visualised with the WesternBright™ Quantum Western Blotting Detection Kit (Advansta) and densitometry analyses performed using IMAGEQUANT software (Molecular Dynamics).

### Ascorbate peroxidase activity assay

To investigate whether differential accumulation of oxidative stress with time/age is caused by impaired antioxidant defence system responses, we compared the activity of the cytosolic ascorbate peroxidases (APX), which protects cells against H_2_O_2_ accumulation^52^. APX activity was measured in the juvenile and adult leaves from WT and *ftsh4* mutant plants of different ages.

In all, 50 mg of leaves were homogenised in liquid nitrogen and suspended in 200 μL of 100 mM potassium phosphate extraction buffer (PBS), pH 7.0, containing 5 mM ascorbate and 1 mM EDTA at 4 °C. After centrifugation at 14, 000 g for 30 min at 4 °C, the supernatant was used for zymograms^53^,^54^. Native electrophoretic separation was performed on 12% polyacrylamide gels^55^,^56^. After electrophoresis, gels were rinsed (3×) for 10 min in 50 mM PBS, pH 7.0, containing 2 mM ascorbate, then soaked in 50 mM PBS, pH 7.0, containing 4 mM ascorbate and 1 mM H_2_O_2_ for 20 min, then rinsed in distilled water, and lastly incubated in 50 mM PBS, pH 7.8, containing 14 mM TEMED and 2.45 mM NBT for 10–30 min. APX activity manifests as a bright bands in uniformly stained gel.

### SAM *in vivo* fluorescent analyses

H_2_O_2_ was measured using 2′,7′-dichlorodihydrofluorescein diacetate (DCFH-DA)^57^ on hand-dissected juvenile and adult vegetative shoot apices (SAM and the youngest primordia) from WT and *ftsh4*-*1* plants grown under LD at 30 °C. Longitudinal sections of shoot apices were prepared with a razor and incubated for 40 min at 30 °C in 50 μM DCFH-DA dissolved in 100 mM of PBS, pH 7.4. The images from at least 10 individual plants from biological repeats were performed using confocal laser scanning microscope (CLSM, see *Microscopy* section). As a positive control, WT samples were chemically stimulated to produce H_2_O_2_, and the strong accumulation of the fluorescent signals was detected (Supplementary Fig. 4).

In the transgenic lines, juvenile and adult vegetative shoot apices from the DIPS*-GFP* reporter line in WT and *ftsh4*-1 backgrounds grown under LD at 30 °C and 22 °C were hand-sectioned as described above. RAM was analysed in roots of 8 day old plants. To analyse specifically the root growth, plants of both genotypes were first grown for 3 days in 22 °C and then transferred for further 5 days to 30 °C. CLSM was used to localise GFP signal on the longitudinal median sections of shoot apices from at least 10 individual plants from each transgenic line and growth condition, in biological repeats. The percentage of the area showing depletion of mitochondrial GFP signal was calculated using AxioVisionLE 4.2 software. Chlorophyll autofluorescence was detected using CLSM on a subset of hand-sections of DIPS-*GFP* WT and *ftsh4-1* lines grown at 30 °C.

### Microscopy

The plant material and prepared slides were examined by the techniques specified above using the following equipment: (a) a digital camera; (b) a stereomicroscope with a digital camera and InSight software (DeltaPix InSight Inc.); (c) an epi-fluorescence microscope with bright-field optics equipped with a digital camera and CellˆB software (Olympus Optical); and (d) an inverted CLSM.

Hoechst 33342 fluorescence was observed under the epi-fluorescence microscope with the excitation wavelength of 350 nm (UV). In CLSM, excitation and emission wavelengths for the different measures were: DCFH-DA 473/490 to 590 nm, DIPS-*GFP* lines 473/490 to 590 nm, chlorophyll autofluorescence 635/655 to 755 nm. Imaging was performed with a ×60 lens. GFP detection and chlorophyll autofluorescence used the multitrack mode with line switching between channels; their excitation and emission spectra did not overlap. CLSM settings (e.g. laser power, scanning conditions) were maintained for all genotypes. For a subset of CLSM images, Z-axis projections from 2–3 single scans were made.

## Additional Information

**How to cite this article**: Dolzblasz, A. *et al*. The mitochondrial protease AtFTSH4 safeguards *Arabidopsis* shoot apical meristem function. *Sci. Rep.* **6**, 28315; doi: 10.1038/srep28315 (2016).

## Supplementary Material

Supplementary Information
