# Supplementary material for: The mitochondrial protease AtFTSH4 safeguards Arabidopsis shoot apical meristem function
Source: Sci Rep. 2016 Jun 20;6:28315. doi: 10.1038/srep28315 (PMC4913265; doi:10.1038/srep28315)
Supplement: Supplementary Information [file srep28315-s1.pdf]

Supplementary information SI for the manuscript:

The mitochondrial protease AtFTSH4 safeguards *Arabidopsis* shoot apical meristem function

Alicja Dolzblasz[1,*], Elwira Smakowska[2,3], Edyta M. Gola[1], Katarzyna Sokołowska[1], Marta Kicia[2,4], Hanna Janska[2*]

[1] Department of Plant Developmental Biology, Institute of Experimental Biology, Faculty of Biological Sciences, University of Wroclaw, Wroclaw, Poland

[2] Department of Cellular Molecular Biology, Faculty of Biotechnology, University of Wroclaw, Wroclaw, Poland

[3] Current address: Gregor Mendel Institute (GMI), Austrian Academy of Sciences, Vienna Biocenter (VBC), Vienna, Austria

[4] Current address: Department of Biology & Medical Parasitology, Wroclaw Medical University, Wroclaw, Poland.

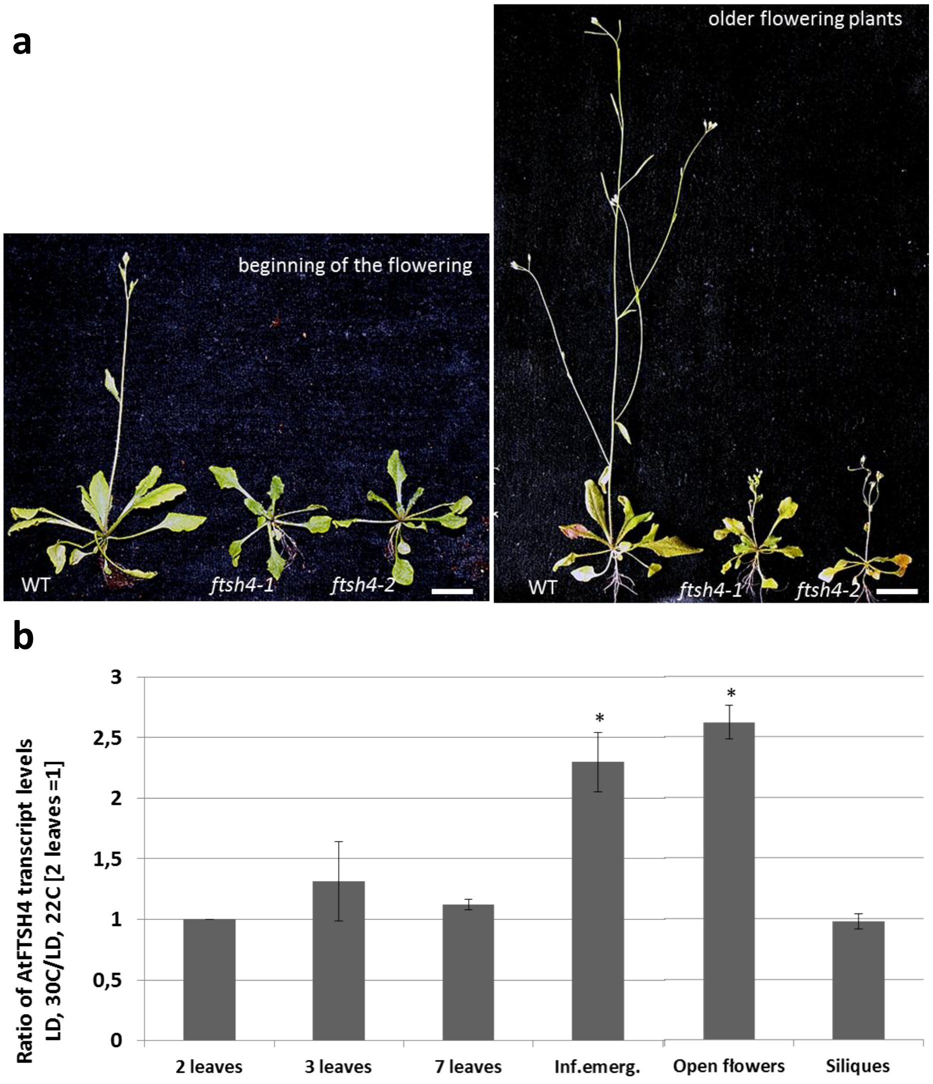

Supplementary Figure 1. The growth retardation of *ftsh4-1* and *ftsh4-2* mutant plants grown in LD, 30°C and *AtFTSH4* transcript level comparison.

- **(a)** Comparable pictures of two *ftsh4* mutant alleles (*ftsh4-1*, *ftsh4-2*) and wild-type plants at successive generative developmental stages (when WT plants have around 6 cm height inflorescence and when they are fully mature) during the growth in 30°C. The stages are depicted. Both *ftsh4* mutant alleles are characterized by growth retardation. Scale bar: 1 cm
- **(b)** Ratio of the *AtFTSH4* transcript levels analysed during vegetative and generative development of WT plants grown under LD, 30°C to the ones grown under LD, 22°C. Ratio (or fold change) was estimated for the following tissue samples: rosette leaves (2nd, 3th, 7th) sampled during the vegetative stage of development, 7th leaf dissected after flowering ("infl. emergence"), flowers and siliques. Abundance of *AtFTSH4* transcripts is relative to the youngest tissue samples (2 leaves). Mean values ± SD from three experiments are shown. The asterisks indicate significant differences to the 2-leaf sample at $p < 0.05$.

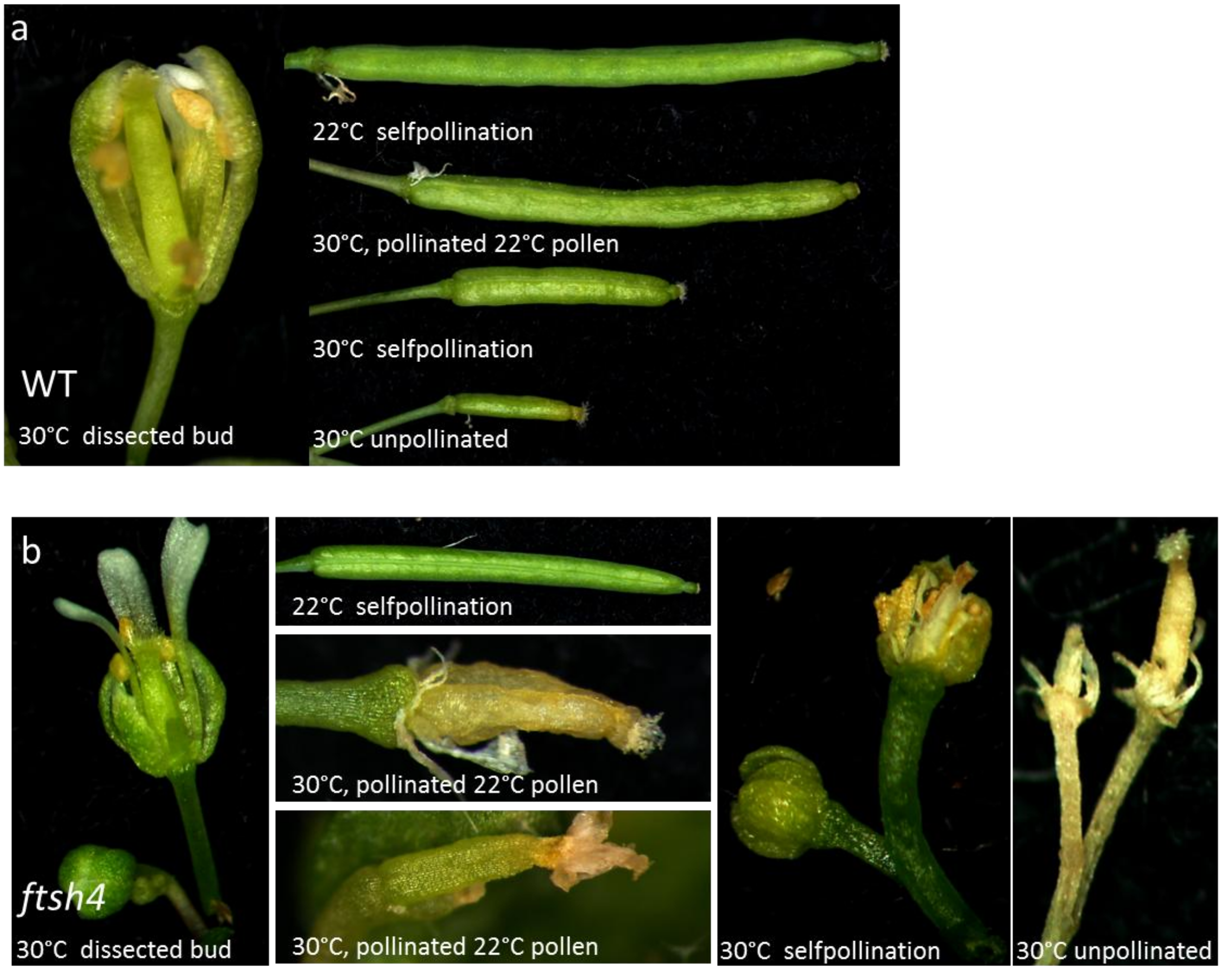

Supplementary Figure 2. The maternal/paternal defect during fertilization in 30°C conditions.

(a) Representative flower bud of a WT plant grown in 30°C showing a carpel and a comparison of siliques formed in a cross-pollination experiment. The carpel either was left unpollinated (a negative control when all the stamens were removed), was left to selfpollinate (the stamens were left to proceed with a pollination process) or was pollinated, after the removal of its own stamens, with a pollen taken from a wild-type plant grown in 22°C; and as a control is shown a silique which was formed on a plant grown in 22°C after a self pollination. The silique formed with use of a pollen taken from a 22°C grown plant fully resembles a silique on plants grown in 22°C.

(b) Representative flower bud of a *ftsh4* mutant plant grown in 30°C showing a carpel and a comparison of siliques formed in a cross-pollination experiment. The carpel either was, as with WT plants, left unpollinated (a negative control when all the stamens were removed), was left to self pollinate (the stamens were left to proceed with a pollination process) or was pollinated, after the removal of its own stamens, with a pollen taken from a wild-type plant grown in 22°C; and as a control is shown a silique which was formed on a plant grown in 22°C after a self pollination. *ftsh4* mutant plants grown in 30°C are always unable to form siliques, even with use of a pollen taken from a 22°C grown plant and dry off pointing to maternal defects.

a

	time of transfer	Number of axillary stems in categories (%)			N
		0-2	3-4	5-7	
WT	1	100	0	0	20
	2	91,7	8,3	0	12
	3	88,9	11,1	0	9
	4	93,8	6,2	0	16
	5	88,9	11,1	0	18
ftsh4	1	72,2	22,2	5,6	18
	2	70,6	29,4	0	17
	3	47,8	30,4	21,8	23
	4	52,4	28,6	19	21
	5	90,5	9,5	0	21

b

	time of transfer	Number of axillary stems in categories (%)			N
		0-2	3-4	5-7(8)	
WT	1	92,9	7,1	0	14
	2	92.3	7.7	0	13
	3	100.0	0	0	17
	4	84.2	15.8	0	19
	5	81,5	18,5	0	27
ftsh4	1	72.2	22.2	5.6	18
	2	60.0	40.0	0	10
	3	68.4	31.6	0	19
	4	85.7	14.3	0	7
	(4)	(35.3)	(35.3)	(29.4)	17
	5	100.0	0	0	15

c

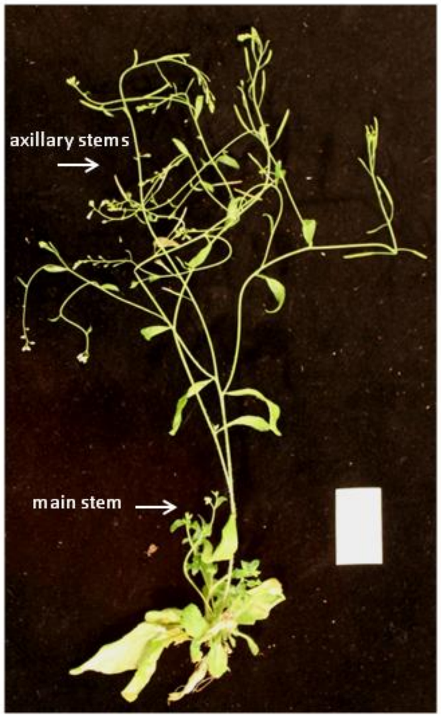

Supplementary Figure 3. Axillary stem formation depending on the time grown at 30°C (relates to Figure 2 and Figure 3).

(a) The number of plants (shown in %) grouped according to the denoted number of axillary stems: 0-2, 3-4 or 5-7(8) axillary stems per plant. Numbers from 1 to 5 specify the timing of transfer from LD 22°C to 30°C: 1- plants growing continuously at 30°C; transferred at the stage of: 2- two leaves, 3- juvenile, 4- adult, 5- after bolting. N – number of plants analysed.

(b) The number of plants (shown in %) grouped according to the denoted number of axillary stems: : 0-2, 3-4 or 5-7(8) axillary stems per plant. Numbers from 1 to 5 specify the timing of transfer from LD 30°C to 22°C: 1- plants growing continuously at 22°C; transferred at the stage of: 2- two leaves, 3- juvenile, 4- adult, 5- after bolting. In brackets () are shown data from plants transferred at the adult phase (4) and analysed after a prolonged time (2-3 weeks) in the standard 22°C conditions. N – number of plants analysed.

(c) The representative phenotype of a *ftsh4* mutant plant removed from the mild temperature stress growth conditions of 30°C at the adult stage (4) (already committed to flowering) after around 2-3 weeks of growth in a standard 22°C. The main inflorescence shoot (main stem pointed by an arrow) terminates its growth still relatively quickly but the axillary stems (pointed by an arrow) develop in excess and are able to form normally looking siliques.

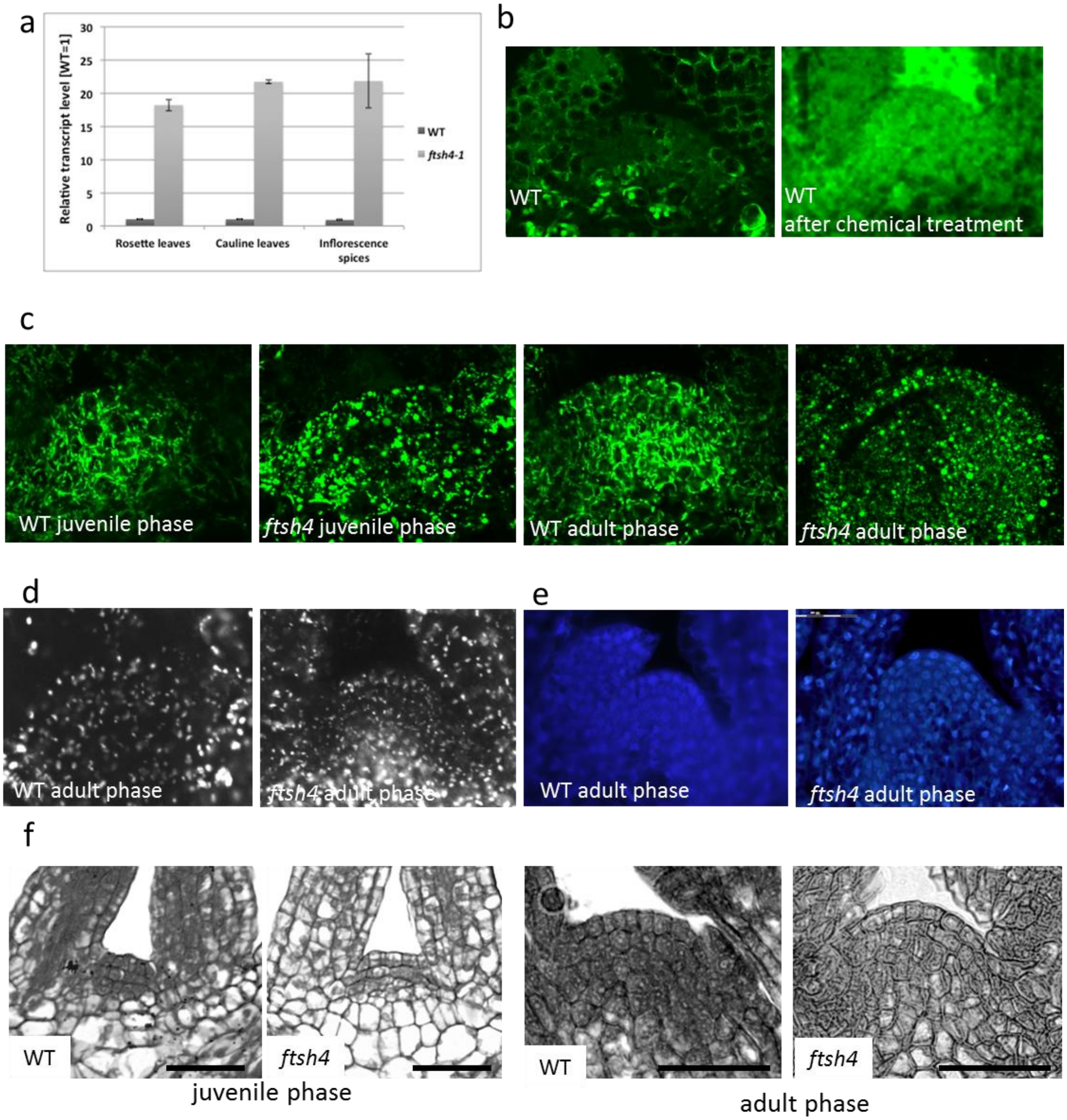
a
Relative transcript level [WT=1]
30
25
20
15
10
5
0
Rosette leaves
Cauline leaves
Inflorescence spices
WT
ftsh4-1
b
WT
WT
after chemical treatment
c
WT juvenile phase
ftsh4 juvenile phase
WT adult phase
ftsh4 adult phase
d
WT adult phase
ftsh4 adult phase
e
WT adult phase
ftsh4 adult phase
f
WT
ftsh4
juvenile phase
WT
ftsh4
adult phase

Supplementary Figure 4. Additional information about meristem structure and stress response.

- **(a)** The comparison of *UPOX* gene transcript levels in different, both vegetative and generative organs (rosette leaves, cauline leaves, inflorescence shoot apices) of *ftsh4* mutants and WT plants grown in 30°C prior to the growth cessation. In all cases the level was increased in mutant plants. Analysis performed by quantitative real-time PCR.
- **(b)** The H_2O_2 *in vivo* accumulation in the meristems of adult WT plant, measured with use of fluorescent probe CH-H_2DCFDA, after oxidative stress induction by the application of the Rotenone and Antimycin A solution (right) to the WT adult SAM, in comparison to the untreated sample (left).
- **(c)** The comparison of mitochondria distribution and morphology *in vivo* in the meristems of juvenile and adult WT and *ftsh4* mutants, grown in 22°C, as measured with use of transgenic lines carrying mitochondrial-green fluorescent protein (GFP) marker (DIPS-GFP, mitoTraker-GFP). The WT plants (WT;35S:Mt-GFP) are characterised by small mitochondria evenly distributed throughout the meristem, whereas in *ftsh4* mutant plants (*ftsh4*;35S:Mt-GFP) already form mitochondria aggregates, additionally small areas of the meristem are seemingly devoid of mitochondria.
- **(d)** The proplastids distribution and morphology *in vivo* in the meristems of adult WT and *ftsh4* mutants (transgenic lines DIPS-GFP (mitoTraker-GFP), grown in 30°C.
- **(e)** The nuclei distribution and morphology *in vivo* in the meristems of adult WT and *ftsh4* mutants, grown in 30°C, as visualised with Hoechst staining.
- **(f)** The comparison of WT and *ftsh4* mutants meristem structures at the juvenile and adult stages of development. Both genotypes had the same meristem structure.

  Scale bars: (c) 20µm, (f) 50µm

Supplementary Table 1. Sequence of oligonucleotides used for qRT-PCR.

	**Gene name**	**Forward primer**	**Reverse primer**
**Reference gene**			
	PP2A	TTGGTGCTCAGATGAGGGAGAG	TTCACCAGCTGAAAGTCGCTTAG
**Analysed genes**			
	FTSH4	GACCCCAAAAGGTTCACTCG	AGCCTTAAGTACCTTTGACATG
	UPOX1	ATCATCATGCAGGAAGAGGGTG	GAAGCTCCCGAATATCTTGTCC
	WUS	AACCAAGACCATCATCTCTATCATC	CCATCCTCCACCTACGTTGT
	CLV3	GTTCAAGGACTTTCCAACCGCAAGATGAT	CCTTCTCTGCTTCTCCATTTGCTCCAACC
